# The effects of false feedback on state memory distrust towards commission and omission and recognition memory errors

**DOI:** 10.1098/rsos.251045

**Published:** 2025-09-03

**Authors:** Yikang Zhang, Henry Otgaar, Robert A. Nash, Chunlin Li

**Affiliations:** ^1^ Department of Criminology, Max Planck Institute for the Study of Crime, Security and Law, Freiburg, Germany; ^2^ Clinical Psychological Science, Faculty of Psychology and neuroscience, Maastricht University, Maastricht, The Netherlands; ^3^ Faculty of Law and Criminology, KU Leuven, Leuven, Belgium; ^4^ Aston University, Birmingham, UK

**Keywords:** memory distrust, misinformation, response criterion, commission errors, omission errors

## Abstract

Memory distrust, the subjective appraisal of one’s memory functioning, comprises two aspects: distrust over omission errors (e.g. forgetting) and distrust over commission errors (e.g. falsely remembering). Although these aspects have been studied, how they relate to memory validation (e.g. forming autobiographical beliefs) and memory reporting remains unclear. In this study, we experimentally examined how metacognitive appraisals influence memory validation and errors in memory reporting. Participants (*N* = 622, *M*_age_ = 38.67, s.d._age_ = 12.23) completed a memory task where they received inaccurate feedback about a tendency to make either commission errors, omission errors or no feedback. They then performed a second recognition task. Compared to the control group, those who received feedback suggesting a tendency to make commission errors showed a shift towards a more conservative response criterion. In contrast, those who received feedback indicating a tendency to make omission errors shifted towards a more liberal criterion. However, manipulation checks did not confirm that our manipulations affected state memory distrust as expected, and we did not find sufficient evidence that the effect of feedback operated through changes in state memory distrust. Possible explanations and future directions are discussed.

## 1. Introduction

In most instances of remembering, the person trusts that the recollected event truly happened in the past. Such event representations, which encompass both vivid recollection and a firm belief in the event’s occurrence, are referred to as believed memories [1]. However, there are other types of event representations in addition to believed memories. For some events, people can hold strong beliefs about their occurrence without any recollections about them, such as the celebration of their first birthday (believed-but-not-remembered events). More interestingly, in some instances, people can recollect certain events vividly but do not believe the events have happened in the past. The latter phenomenon is known as non-believed memories [2,3]. Cases where the recollection and belief of a memory diverge reveal that autobiographical recollection and belief of an event are two related but distinct constructs [1–4].

The dissociation between recollection and belief can also be observed in other ways. Scoboria *et al*. [1] reported that memory characteristics that predict recollection ratings well (e.g. perceptual, re-experiencing, emotion intensity, event specificity) did not predict belief ratings and vice versa (e.g. plausibility judgement). Furthermore, studies on non-believed memory showed that autobiographical beliefs can be altered relatively easily—more so than can recollections—in response to social information contradicting one’s memories [5–9]. In particular, the credibility of social information influences the formation of false autobiographical beliefs but not the recollective features [1]. On the other hand, in the course of forming false memories, false beliefs can be created more easily relative to false recollections [10,11]. People who start to falsely believe a suggested event may then utilize general scripts for similar events, or memory details of other episodes, to develop their recollections [11].

Blank [12] posited that these recollection-belief divergences, such as non-believed memories or believed-not-remembered events, arise from normal, healthy metacognitive monitoring and control processes that balance recollections and reality constraints. Specifically, Blank [13] proposed a theoretical framework of remembering that distinguishes memory, belief and the communication of memory (e.g. memory reporting) and elaborates on the role of social influence in remembering. First, memory traces are activated through either internal process (e.g. intentional searches) or external cues (e.g. verbal prompts). The second stage involves a validation process in which the validity of the memory is inferred from both the retrieved internal information and from external information (e.g. physical evidence such as photos or social feedback) to form a (memory) belief. Finally, the rememberer decides whether or not to communicate and how to communicate the belief to others. In this view, an autobiographical belief is the summative evaluation of the truth status of the remembered events at the time of retrieval (see also [1,3,14]). Notably, since the information available could change in each instance of remembering, the truth status assigned to the events could therefore also be revisited and substantially altered, possibly resulting in the change of memory reports, such as the retraction of allegations of sexual abuse [12,15].

Expanding this theoretical framework, recent research brings to the discussion the roles of beliefs and appraisals in the process of memory validation (e.g. [16–18]). More specifically, researchers have argued that memory distrust, defined as the sceptical perception or appraisal of one’s own memory functioning, contributes to the formation of false memory and non-believed memory [16,18]. The term ‘memory distrust’ was first coined by Gudjonsson & MacKeith [19] to describe a phenomenon in which interviewees in a forensic context develop profound distrust towards their memories due to inappropriate investigation practices. Subsequently, researchers expanded the concept of memory distrust to denote a more general and stable appraisal of one’s memory functioning [16,20]. As of now, there exist two validated scales measuring trait memory distrust: the adapted Squire Subjective Memory Questionnaire (SSMQ) [20,21] focuses on people’s beliefs about their susceptibility to memory omission errors (i.e. forgetting), whereas the Memory Distrust Scale (MDS; [16]) measures beliefs about susceptibility to commission errors (i.e. falsely remembering events which never happened).

Zhang *et al*. [17,18] argued that individuals vary in how much they trust their recollections when comparing them to external information. Specifically, people with high memory distrust (versus low memory distrust) would give less credence to their recollections and would, in turn, lower their memory beliefs. Building on this premise, Zhang *et al*. [17,18] examined the relationship between memory distrust (as measured by the SSMQ) and non-believed memories, finding that people with high memory distrust were more likely to report non-believed memories than those with low memory distrust. Similarly, using an indirect recall method, in which participants were given cues to recall past events and then rated their recollections and beliefs about those events, Nash *et al*. [16] found that people who reported non-believed memories, compared with people who did not, scored higher on average on memory distrust (as measured by the MDS). Furthermore, in one study, individuals with high or low memory distrust differed in their chosen strategies for verifying personal memories [22]. In Zhang *et al*.’s [22] research, people with high (versus low) memory distrust, when confronted with the possibility of erroneous memory, were more likely to use low-cost but less reliable verification strategies (e.g. seeking information from other people), thus exhibiting a greater ‘cheap-and-easy strategy’ bias [23,24], and they seemed less likely to rely on their recollections for making belief judgements, even in the absence of external information [25].

A more nuanced picture of the relationship between memory distrust and memory appraisal was revealed through analyses using the signal detection theory (SDT) [26]. SDT is a framework designed to analyse people’s ability to differentiate between signal and noise (i.e. sensitivity or discriminability) and their thresholds for deciding that a given stimulus is a signal (i.e. response criterion), in decision-making processes such as recognition memory. For example, some people may have a very conservative response criterion and therefore only judge a stimulus as signal when the evidence is strong, resulting in them making few false alarms but also having relatively poor correct recognition of the signal (i.e. hits). Others, in contrast, might respond more liberally, requiring more moderate evidence for judging a stimulus as signal and, therefore, making more hits and more false alarms. Zhang *et al*. [27] reported a positive association between participants’ scores on the MDS (but not SSMQ) and the response criterion index *β*, with higher values indicating a more conservative response bias (i.e. a bias towards judging stimuli as new in a recognition task). However, in another study [25], higher memory distrust as indexed by the MDS was negatively associated with *β,* whereas higher memory distrust as indexed by the SSMQ was positively associated with *β*. Put differently, in one study, people who believed that they were susceptible to certain memory errors exhibited response biases that aligned with those subjective beliefs [25], whereas in another study [27], people who believed themselves to be susceptible to certain memory errors exhibited response biases that seemed to compensate for that susceptibility.

Two major differences in the design may have caused these inconsistent results, as argued by Zhang *et al*. [25]. First, in study 2 of Zhang *et al*. [27], participants were given (correct or incorrect) feedback allegedly from another participant after each judgement. Second, there was a cash incentive for high performers in the memory task of that study. Both features were absent in Zhang *et al*. [25]. Peer feedback and performance incentives might lead participants to adjust their responses. They may do so by applying subjective memory appraisals to their judgements. For example, if a participant thinks that they often make commission errors, they should then have a higher threshold to make an ‘old’ judgement in this task, and in the absence of incentive, this participant may rely heavily on recollection to make this judgement [12], as reflected in the positive associations of memory distrust with recollection and with *β* in Zhang *et al*. [25]. But in the presence of an incentive, a participant who thinks they often make commission errors might aim to improve their accuracy by adopting a more conservative response criterion. Taken together, accumulating evidence leads us to propose that under specific conditions, people apply their beliefs about their subjective memory functioning to calibrate their evaluations of their recollections.

So far, the limited work on the role of memory distrust in the memory validation process considered only trait memory distrust (e.g. [25,27]), which complicates the picture as it is associated with the objective functioning of memory. That is, trait memory distrust may influence memory performance through various mechanisms. First, given that trait memory distrust is associated with objective memory functioning, we can expect people who are high (versus low) on memory distrust to perform worse in memory tasks (i.e. lower sensitivity [25]; but see [28] for a different result). Second, in situations where individuals reflect upon their subjective memory functioning and calibrate their memory validation based on it, we would expect memory distrust to have a different impact on memory performance, e.g. response bias. Furthermore, the moderation effect of memory distrust on recollection-belief correspondence is complicated by the fact that memory distrust is associated with both belief and recollection ratings [25].

To single out the potential calibration effects of memory distrust on memory validation, the current study aims to experimentally manipulate participants’ concern over making omission errors or commission errors and examine the effect of this manipulation on subsequent memory reporting, including recognition, recollection and belief judgements. We hypothesized that with an incentive to be as accurate as possible, participants who are told that they often make commission errors will adjust their response criterion and show a shift towards a more conservative response bias. On the other hand, participants who are told that they often make omission errors will show a shift towards a more liberal response bias. The idea that feedback can induce criterion shifts is by no means novel. Research has shown that biased positive feedback on incorrect old classification of new items (i.e. false alarm), or incorrect new classification of old items (i.e. miss), caused participants to shift towards a more lax or conservative response criterion, respectively, without them being aware of the biased nature of the feedback [29,30]. Despite the similarity, the focus of the current study is not to examine the incremental criterion shift based on trial-by-trial positive feedback, but rather, a more general appraisal of one’s memory functioning that is influenced by negative feedback.

## 2. Methods

All study materials are available at the Open Science Framework (OSF; (https://osf.io/8qbkn/?view_only=a663f2e3619545edafe86b0aee885603). The present study received ethical approval from the Ethics Review Committee Psychology and Neuroscience (ERCPN) of Maastricht University (reference: ERCPN-OZL_246_167_12_2021_S5). The accepted stage 1 manuscript can be accessed at https://osf.io/x69qt.

## 3. Design

The study used a between-participant design with three conditions (feedback: omission versus commission versus no feedback).

## 4. Participants

### 4.1. Sample size justification

#### 4.1.1. Expected effect size of criterion shift

We expected the effect of the memory distrust manipulation on criterion shift to be smaller than the effect of explicit instructions to respond liberally or conservatively (*c*_*a* conservative_ = 0.34, *c*_*a* liberal_ = -0.50, *c*_*a* diff_ = 0.84 [31]) but larger than the effect of implicit biased feedback (experiment 2: *c*_*a* conservative_ = 0.39, *c*_*a* liberal_ = 0.02, *c*_*a* diff_ = 0.37; experiment 3: *c*_*a* conservative_ = 0.19, *c*_*a* liberal_ = 0.05, *c*_*a* diff_ = 0.14 [29]). We therefore set a conservative expected effect size as a difference of *c* = 0.15 between the omission condition and the control condition and between the control and commission condition (in between experiments 2 and 3 in [29]).

#### 4.1.2. Smallest effect size of interest of criterion shift

We consider the following data pattern to be the smallest effect size of interest (SESOI) in the current experimental set-up: the top 25% of participants who are most receptive to the memory distrust (omission) manipulation will make one more hit response and one more false alarm response compared to their control counterparts in the current experiment. Similarly, the top 25% of participants who are most receptive to the memory distrust (commission) manipulation will make one less hit response and one less false alarm response compared to their control counterparts.^1^ The justification behind one hit and one false alarm comes from the idea that the remembrance of just one (false) detail can have practical value (e.g. misremembrance of the face of a culprit). Using the data from study 2 of Zhang *et al*. [27] as the control condition and the expected differences between conditions, we created a synthetic dataset and calculated the SDT indices (see table 6). In the synthetic dataset (see table 6), the average difference of response criterion c is 0.06 between the feedback-omission and control or between control and the feedback commission with an s.d. of 0.30 (i.e. Cohen’s *d* = 0.06/0.30 = 0.2). With the same s.d. assumed, our expected effect size would translate to a difference of Cohen’s *d* = 0.50.

#### 4.1.3. Expected effect size of state memory distrust

Given that in Dudek & Polczyk [32], the difference of memory distrust between the experimental group and control group was close to that of Cohen’s *d* = 1.0, we consider the expected effect size of our manipulation on state memory distrust to be Cohen’s *d* = 1.0.

#### 4.1.4. Smallest effect size of interest of state memory distrust

Assuming that memory distrust is the underlying mechanism of response criterion change and that the correlation between state memory distrust and response criterion c is at least *r* = 0.25,^2^ this requires that the strength of the manipulation to be no smaller than Cohen’s *d* = 0.2/0.25 = 0.8 for comparisons between memory distrust conditions and the control condition (calibration [33]). That is, state memory distrust towards omission should be 0.8 s.d. higher in the feedback-omission condition than in the control condition. Similarly, the state memory distrust towards commission should be 0.8 s.d. higher in the feedback-commission condition than in the control condition.

#### 4.1.5. Power analyses

We performed simulation-based power analysis for minimal effect testing and equivalence testing (see [34] for the tutorial) for the pairwise comparisons of response criterion and state memory distrust between the (i) control and omission conditions and (ii) control and commission conditions. Specifically, the minimal effect testing calculated the percentage of simulations wherein the lower bound of an 80% confidence interval (CI) of the effect was greater than our SESOI (i.e. *c*_diff_ = 0.06, raw score difference of state memory distrust = 1.6 scale-points). The equivalence testing calculated the percentage of simulations wherein the 80% CI fell within the two equivalence bounds (i.e. *c*: (−0.06, 0.06), state memory distrust: (−1.6, 1.6)). This analysis on criterion c showed that when the true effect size is *c*_diff_ = 0.15 (Cohen’s *d* = 0.5), a group size of 100 participants would have 80% power to detect the minimal effect. Looking instead at memory distrust, the analysis showed that when the true effect size on memory distrust is a raw score difference of 2 points (Cohen’s *d* = 1.0), a group size of 210 participants would have 80% power to detect the minimal effect of 1.6. We therefore decided to set the minimum number of participants as 210 per group based on the requirement of state memory distrust (https://osf.io/5x43j).

For the analyses on criterion c, a group of 210 participants would afford 96% power to detect the minimal effect of *c*_diff_ = 0.06. With this group size, there is a 54% probability that the 80% CI would fall between the equivalence bounds (−0.06, 0.06) when the true effect is 0. For the analyses on state memory distrust, with the same group size and when the true effect is 0, the 80% CI will fall between the equivalence bounds (−1.6, 1.6) almost 100% of the time (https://osf.io/hxb6c). For both criterion c and state memory distrust, when the true effects are zero, with a sample of 210, in 0 out of 1000 simulations, the 80% CIs are above the minimal effects of interest (i.e. a false positive).

Sensitivity analysis with G*Power 3.1 [35] showed that a sample of 630 (210 × 3) would allow us to detect a slope of 0.015 criterion (c) units/Likert unit of state memory distrust (commission or omission) in a linear regression examining the association between state memory distrust and response criterion c (*α* = 0.05 and 1 − *β* = 0.80; see appendices— sensitivity analysis protocol).

We recruited 787 participants using Connect (https://connect.cloudresearch.com/), among whom, 653 participants completed both sessions. After excluding three participants who had participated in similar studies and 28 participants who questioned the validity of the performance feedback, per our preregistration, the final sample consisted of 622 participants (*n*_women_ = 306, *n*_men_ = 299, *n*_nonbinary_ = 2, *n*_other_ = 1, *n*_missing_ = 14; *M*_age_ = 38.67, s.d._age_ = 12.23). The only inclusion criteria were that participants should be aged 16 or above, have normal (corrected) vision and be fluent English speakers. Participants could only sign up for and complete the experiment using laptops or PCs (i.e. no tablet or mobile answering), and they received $1.20 for the first session (approx. 10 min) and $1.80 for the second session (approx. 18 min) 1 day later. Participants whose memory task performance was among the top 10% also received an additional bonus of $3 (see the procedure below).

## 5. Material and procedure

### 5.1. Stimuli for the memory task

To increase the generalizability of our findings across a wider range of stimuli, but also to minimize the potentially unnecessary emotional impact on participants, a total of 80 valenced (valence ≥3 and arousal ≤5 on 7-point scales) colour images were selected from the open affective standardized image set (OASIS [36]), an open-access stimulus set depicting a broad spectrum of natural or social situations (e.g. pets, people, buildings or car accidents). We then randomly divided these 80 scenes into two 40-scene blocks that were used either as old (i.e. appearing in encoding) or new stimuli (i.e. only appearing in tests as fillers). Each block was then subdivided into two sub-blocks of 20 scenes that were tested either during test 1 or test 2. Using Kurdi *et al*.’s [36] norming data, we performed two-way between-subject ANOVAs to ensure that there was no statistically significant difference in the mean level of arousal and valence ratings between different blocks and old versus new scenes (see table 7 in the appendix).

Forty scenes were presented during encoding (hereafter referred to as old scenes), and another 40 scenes were used as fillers for the first and second recognition tests. Old scenes and new scenes were counterbalanced between participants. That is, each scene was an old scene for half of the participants while being a new scene for the other half of the participants. Designated old scenes and new scenes were further randomly divided into two blocks (*n* = 20). For the first recognition task, one block of old scenes and one block of new scenes as fillers were randomly selected and presented to participants, leading to a recognition test containing 40 scenes. In the second recognition task, participants were presented with the remaining block of old scenes and the remaining block of 20 new scenes. The order of the blocks is thus also counterbalanced.

### 5.2. Manipulation check

Two statements measuring state memory distrust were completed using a 10-point Likert scale (from 1 = extremely disagree to 10 = extremely agree). The first statement is ‘At this moment, if I am asked to retrieve something from memory, I think I likely would remember things that did not happen’ (state memory distrust towards commission). The second statement is ‘At this moment, if I am asked to retrieve something from memory, I think I likely would forget things that happened’ (state memory distrust towards omission). Half of the participants answered the manipulation checks before the second test, and half of the participants answered the manipulation checks after the second test. This random assignment was intended to examine whether the expected effect is influenced by the timing of the manipulation check, which could, in principle, make the feedback on commission /omission errors more salient. For example, it could be that there is a stronger effect of feedback in the manipulation-check-first conditions than in the memory-test-first conditions.

### 5.3. Trait memory distrust

As stated earlier, previous research showed that aspects of trait memory distrust are differently associated with response bias and memory performance. We therefore also include measures of trait memory distrust in the current study, in an attempt to further examine the associations. The SSMQ [21] adapted by van Bergen *et al*. [20] has 18 items (e.g. ‘my ability to pay attention to what goes on around me’ is from −4 = *Disastrous* to 4 = *Excellent*), measuring distrust towards making omission errors. The MDS [16] consists of 20 items (e.g. ‘I am sometimes uncertain whether an event that I recall really happened to me, or whether I saw it on TV or in a movie’ from 1 = *Strongly disagree* to 7 = *Strongly agree*), measuring distrust towards commission errors. To ease the comparison of results, after establishing internal consistency of the SSMQ and the MDS in the current sample (Cronbach’s *α* = 0.95 and 0.97, respectively), we reverse-coded the SSMQ and then calculated the mean of all items for the two scales, so that a higher mean score in both scales reflects a higher level of memory distrust. Following this transformation, the two scales were positively correlated in the current sample, *r* = 0.46, *p* < 0.001, 95% CI (0.40, 0.51).

### 5.4. Procedure

#### 5.4.1. Session 1

**Figure 1.**
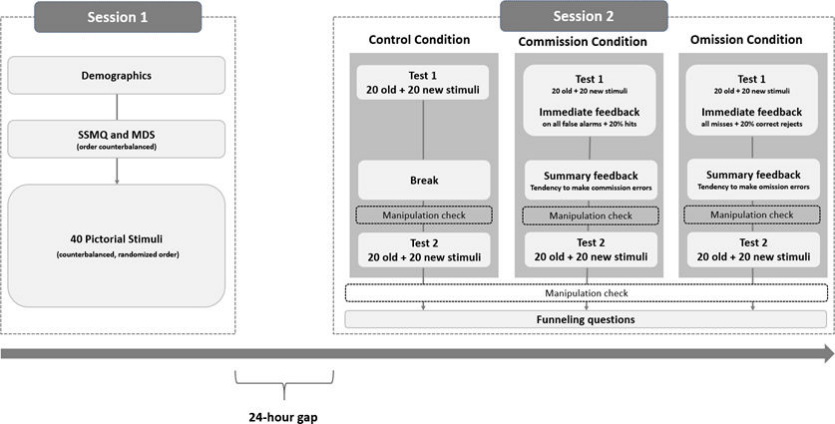
Flowchart of the study procedures. Note. Dashed lines surrounding manipulation check indicate that the order of manipulation check (before and after test 2) was randomized.

After reading the information letter and giving informed consent, participants first answered demographic questions about their age, gender and education level, followed by the SSMQ and the MDS in counterbalanced order. Embedded in the two scales, we included three attention checks asking participants to choose a specific answer for that item (e.g. for this item, please choose ‘strongly agree’). Participants then viewed 40 scene images, one at a time and in randomized order. Each scene was presented for 3 s with an inter-stimulus interval of 1 s. At the end of session 1, participants were reminded to sign up for and complete session 2 at the same time the next day (see figure 1 ). In the current study, we expected to complete session 1 data collection within 3 h. If, however, there were not enough participants signing up for the study within a 3 h window, we closed the signup for session 1 after 3 h and ran another cycle of data collection until the planned sample size was met.

#### 5.4.2. Session 2

Twenty-four hours later, session 2 was made available online. Participants then had a 6 h window to sign up for and complete session 2, after which the session was closed. They were first reminded of the tasks involved in the study and informed that they would receive feedback on their incorrect judgements after each of their decisions. The session began with a first recognition task, presenting 20 previously seen (old) scenes alongside 20 new scenes, displayed individually in a random sequence without a time constraint. In this task, participants first indicated whether a scene was old or new (‘Did you see this exact scene in yesterday’s session?’ Options: *yes* or *no*). Regardless of their recognition judegment, participants then assessed their levels of recollection (‘Do you actually remember seeing this exact scene in yesterday’s session?’) and belief in occurrence (‘Regardless of whether or not you remember this scene, do you believe that this scene appeared in yesterday’s session?’) on an 8-point Likert-like scale (Memory: 1 = *No memory at all*, 8 = *Clear and complete memory;* Belief: 1 = *Definitely did not appear*, 8 = *Definitely did appear*). These questions were adapted from [37] and [5].

After making recognition, recollection and belief judgements for each scene, participants were shown feedback on supposedly incorrect recognitions. In reality, the feedback contained both true and false feedback, depending on their assigned condition, to increase the credibility of the feedback manipulation. For participants who were in the feedback-commission condition, they received false feedback on some of the old items that they had correctly identified as old and true feedback on new items they had incorrectly identified as old. For each correctly recognized old item, there was a 20% probability that participants received false feedback that this was actually a new scene. For each incorrectly recognized new item, participants received true feedback that this is a new scene. No feedback on non-identified old items (i.e. misses) was given. Participants who were in the feedback-omission condition received false feedback on some of the new items that they had correctly identified as new and true feedback on old items that they had incorrectly identified as new. For each correctly recognized new item, there was a 20% probability that participants received false feedback that this was actually an old scene. For each incorrectly recognized old item, participants received correct feedback that this was an old scene. No feedback on incorrectly identified new items (i.e. false alarms) was given. For participants in the control group, no feedback was given.

Immediately after the first recognition test, participants in the feedback-commission condition received inaccurate summary feedback claiming that they made several mistakes in the test, misidentifying as ‘old’ several scenes that were not seen in session 1. Participants in the omission error condition received inaccurate summary feedback claiming that they had made several mistakes in the test, misidentifying as ‘new’ several scenes that were actually seen in session 1. Although the majority of participants made both commission and omission errors, consistent with the manipulation during the first test, they only received feedback focusing on one type of error (or no feedback, for participants in the control group). Moreover, irrespective of their actual performance, they were led to believe that they had made more commission/omission errors than the average participant. Accompanying the feedback, we added general descriptions of memory distrust (similar to [32]) to increase the credibility of the feedback (see appendices—feedback script). They were further reminded that participants who ranked in the top 10% of scorers in terms of accuracy in the second memory task would receive an additional monetary bonus ($3) and that it was important to take the memory tasks seriously. The feedback page automatically turned over after 1 min. Participants in the no-feedback condition were given an instruction stating that this was a 1 min break between tests and were reminded of the monetary bonus for top performers, after which the second test commenced. Half of the participants in each condition completed the manipulation checks before the second test.

Then participants completed the second recognition task, in which their memory for the remaining block was tested. All the measures were the same as the first recognition task with no feedback. After the memory tasks, the other half of the participants in each condition completed the manipulation checks. Finally, all participants answered six funnelling questions: (i) ‘To what extent did you take into account your tendency to have memory errors while making recognition judgements in the second memory task?’ (1 = *not at all*, to 7 = *very much*); (ii) ‘To what extent did you find the experiment procedures difficult to understand?’ (1 = *not difficult at all*, to 7 = *very difficult*); (iii) ‘When completing this study, how seriously did you take answering the questions with care? (Your answer will not affect your payment for the experiment)’ (1 = *not serious at all*, to 5 = *very serious*); (iv) ‘Have you seen the materials of this experiment before in other studies’ (Yes or No); (v) ‘What do you think is the purpose of the experiment?’ (Open-ended); (vi) ‘Did you notice any errors in the experiment or do you have any suggestions to improve the experiment?’ (Open-ended).^3^

We then paid participants, debriefed them and paid them the bonus where applicable after the data collection was done. We calculated and ranked the accuracy for each participant within their experimental conditions based on the second recognition task only.

## 6. Data analyses

All data analyses were carried out in R (v. 4.2.2 [38]). All anonymized datasets and coding scripts are available via the OSF (https://osf.io/8qbkn/). Response criterion indices *β* and c were calculated using the *psycho* package [39]. *β* was calculated based on the likelihood ratio of the two distributions (noise and signal), while c represents the distance between the response criterion and the unbiased point, expressed in units of s.d. A higher value of either *β* or c would indicate a greater tendency to recognize stimuli as new instead of old (i.e. a more conservative response criterion). Since *β* is based on a ratio and more likely to violate distribution assumptions than c [25], when the results of the two indices diverged, we gave more weight to c when reaching conclusions. See table 1 for the plans on hypothesis testing.

**Table 1.** Study design table.

question	hypothesis	sampling plan	analysis plan	rationale	interpretation	theory
is the manipulation of memory distrust towards commission effective?	the manipulation of memory distrust towards the commission is effective.	for the single-item measuring state memory distrust towards commission, the expected effect size of the manipulation is Cohen’s *d* = 1.00, given previous research [32]. we will recruit 630 participants (210 in each condition) using Connect. we performed simulation-based power analysis for minimal effect testing and equivalence testing (see [34] for the tutorial) for the pairwise comparisons of state memory distrust. In this analysis, the minimal effect testing calculated the percentage of results wherein the lower bound of 80% CI of the effect is greater than SESOI (raw score difference of state memory distrust = 1.6). The equivalence testing calculated the percentage of results wherein the 80% CI falls within the two equivalence bounds (−1.6, 1.6). when the true effect size on memory distrust is a raw score difference of 2 (Cohen’s *d* = 1), a group size of 210 participants will have 80% power to detect the minimal effect of 1.6.	one-way ANOVA (feedback: commission versus omission versus no feedback) was conducted for state distrust towards commission. only if the lower bound of the 80% CI on the effect size is not lower than Cohen’s *d* = 0.80 (raw score difference of 1.6 assuming a s.d. = 2) for the pairwise comparisons between control and distrust-commission, will we consider the manipulation(s) adequate.	assuming that the correlation between state memory distrust and response criterion c is at least *r* = 0.25, given the minimal effect of memory distrust manipulation on criterion is set to be Cohen’s *d* = 0.20 (see row 3), this requires that the strength of the manipulation to be no smaller than Cohen’s *d* = 0.80.	if the manipulation to increase distrust towards commission errors is successful, we would expect that compared to feedback on omission errors and no feedback conditions, participants in the feedback on commission errors condition will report higher distrust towards falsely remembering. Only if the lower bound of the 80% CI on the effect size is above the minimal effect of interest (raw score difference of 1.6 with a s.d. of 2), will we consider the manipulation adequate. If the 80% CI is within the equivalence bounds (−1.6, 1.6), we will conclude that the manipulation did not reach an adequate strength. If the 80% CI partially overlaps the equivalence region, we will suspend judgement.	NA
is the manipulation of memory distrust towards omission effective?	the manipulation of memory distrust towards omission is effective.	for the item measuring state memory distrust towards omission, the expected effect size of the manipulation is Cohen’s *d* = 1.00, given previous research [32]. we will recruit 630 participants (210 in each condition) using Connect. we performed simulation-based power analysis for minimal effect testing and equivalence testing (see [34] for the tutorial) for the pairwise comparisons of state memory distrust. In this analysis, the minimal effect testing calculated the percentage of results wherein the lower bound of 80% CI of the effect is greater than SESOI (raw score difference of state memory distrust = 1.6). The equivalence testing calculated the percentage of results wherein the 80% CI falls within the two equivalence bounds (−1.6, 1.6). when the true effect size on memory distrust is a raw score difference of 2 (Cohen’s *d* = 1), a group size of 210 participants will have 80% power to detect the minimal effect of 1.6.	one-way ANOVA (feedback: commission versus omission versus no feedback) was conducted for state distrust towards omission. only if the lower bound of the 80% CI on the effect size is not lower than Cohen’s *d* = 0.80 (raw score difference of 1.6 assuming an s.d. = 2) for the pairwise comparisons between control and distrust-omission, will we consider the manipulation(s) adequate.	assuming that the correlation between state memory distrust and response criterion c is at least *r* = 0.25, given the minimal effect of memory distrust manipulation on criterion is set to be Cohen’s *d* = 0.20 (see row 3), this requires that the strength of the manipulation to be no smaller than Cohen’s *d* = 0.80.	if the manipulation to increase distrust towards omission errors is successful, we would expect that compared to feedback on commission errors and no feedback conditions, participants in the feedback on omission errors condition will report higher distrust towards forgetting. Only if the lower bound of the 80% CI on the effect size is above the minimal effect of interest (raw score difference of 1.6 with a s.d. of 2), will we consider the manipulation adequate. If the 80% CI is within the equivalence bounds (−1.6, 1.6), we will conclude that the manipulation did not reach an adequate strength. If the 80% CI partially overlaps the equivalence region, we will suspend judgement.	NA
will the increase in memory distrust towards commission lead to a more conservative response criterion, while the increase of memory distrust towards omission lead to a more liberal response criterion?	the increase in memory distrust towards commission leads to a more conservative response criterion, while the increase in memory distrust towards omission leads to a more liberal response criterion.	we performed simulation-based power analysis for minimal effect testing and equivalence testing (see [34] for the tutorial) for the pairwise comparisons of response criterion. In this analysis, the minimal effect testing calculated the percentage of results wherein the lower bound of 80% CI of the effect is greater than SESOI (*c*_diff_ = 0.06). The equivalence testing calculated the percentage of results wherein the 80% CI falls within the two equivalence bounds, c: (−0.06, 0.06). This analysis on criterion c showed that when the true effect size is *c*_diff_ = 0.15 (Cohen’s *d* = 0.5), a group size of 100 participants will have 80% power to detect the minimal effect. We therefore decide to set the minimum number of participants as 210 per group, which is the larger required sample size.	one-way ANOVA (feedback: commission versus omission versus no feedback) was conducted for response criterion *β* and c. simple effects would reveal that the response criterion is the most conservative in the feedback-commission condition, followed by the control condition. The feedback-omission condition will have the least conservative (most liberal) response criterion. only if the lower bound of the 80% CI on the effect size is equal to or greater than Cohen’s *d* = 0.20 (difference in c is 0.06, assuming an s.d. = 0.30) for the pairwise comparisons (distrust-commission versus control; control versus distrust-omission), will we consider the hypothesis supported.	expected effect size. we expected the effect of memory distrust manipulation on criterion shift to be smaller than explicit instructions to respond liberally or conservatively (*c*_*a* conservative_ = 0.34, *c*_*a* liberal_ = −0.50, *c*_*a* diff_ = 0.84 [31], but larger than implicit biased feedback (experiment 2: *c*_*a* conservative_ = 0.39, *c*_*a* liberal_ = 0.02, *c*_*a* diff_ = 0.37; experiment 3: *c*_*a* conservative_ = 0.19, *c*_*a* liberal_ = 0.05, *c*_*a* diff_ = 0.14 [29]. We therefore set a conservative expected effect size (close to experiment 3 in [29] as a difference of *c* = 0.15 between the omission condition and control condition and between control and commission condition. SESOI. using the data from study 2 of Zhang *et al*. [27] as the control condition and the expected differences between conditions, we created a synthetic dataset and calculated the SDT indices in the synthetic dataset (see table 6), the average difference of response criterion c is 0.06 between feedback-omission and control or between control and feedback commission with a s.d. of 0.30 (i.e. Cohen’s *d* = 0.06/0.30 = 0.2). With the same s.d., the expected effect size translates to a difference of Cohen’s *d* = 0.50.	if our hypothesis is supported, we would expect a main effect of the manipulation. Simple effects would reveal that the response criterion is the most conservative in the commission feedback condition, followed by the control condition. The omission feedback condition will have the least conservative and most liberal response criterion. Only if the lower bound of the 80% CI on the effect size of pairwise comparisons (distrust-commission versus control, control versus distrust omission) is above the minimal effect of interest (criterion c difference of 0.06 with a s.d. of 0.30), will we consider the hypothesis supported. If the 80% CI is within the equivalence bounds (−0.06, 0.06), we will conclude that the hypothesis is rejected and accepted the null hypothesis. If the 80% CI partially overlaps the equivalence region, we will suspend judgement.	if our manipulation is successful yet we do not observe the shift in response criterion, the notion that people calibrate their response criterion based on their memory appraisal will be disconfirmed. The results will have an impact on the frameworks of memory reporting regulation by eyewitnesses.
is state memory distrust towards commission errors associated with a more conservative response criterion while state memory distrust towards omission errors associated with a more liberal response criterion?	state memory distrust towards commission errors is associated with a more conservative response criterion while state memory distrust towards omission errors is associated with a more liberal response criterion.	sensitivity analysis showed that a sample of 630 would allow us to detect a slope of 0.015 c units/Likert unit of state memory distrust (*α* = 0.05 and 1 *− β* = 0.80) in linear regression (see appendices—sensitivity analysis protocol)	linear regression with response criterion c (s.d. = 0.30) as the DV and either state memory distrust towards commission or omission (s.d. = 2.00) as IV. 90% CI was calculated for the regression coefficients to compare against the SESOI.	we expect that the correlation between state memory distrust and response criterion c is at least *r* = 0.25 (raw slope = 0.0375 c units/Likert unit of state memory distrust).	if our hypothesis is supported, we would see that memory distrust towards commission positively predicts β and c, while memory distrust towards omission negatively predicts *β* and c.	even if we receive support that the manipulation affects response criterion, if state memory distrust is not associated with response criterion as we hypothesized, our hypothesis that state memory distrust influences response criterion will be disconfirmed, and the effect of manipulation might be explained by other mechanisms.

### 6.1. Exclusions

We excluded from analyses the data from participants who (i) failed one or more of the attention check questions, i.e. did not respond to the attention checks by selecting the correct or the required answer; (ii) reported being not serious when completing the experiment (score <3); (iii) reported having seen the photo materials before in other studies; or (iv) indicated, in the open question regarding the goal of the experiment, that they distrusted the feedback they received. Two independent coders coded all participants’ open-text responses regarding the latter exclusion criterion based on the gist of their responses. For example, if a participant mentioned gaslighting, manipulation or making people doubt their memories by means of feedback, they were classified as distrusting the feedback (Cohen’s Kappa = 0.77). Any disagreements were resolved during the discussion.

## 7. Results

### 7.1. Primary analysis

#### 7.1.1. Manipulation check: state distrust measures

Two one-way ANOVAs, each with the experimental condition (feedback: commission versus omission versus no feedback) as a between-participant factor, were run for the two manipulation checks (state distrust towards commission and omission) separately. Regarding state distrust towards commission, we failed to detect a statistically significant main effect of the experimental condition, *F*_2, 619_ = 2.75, *p* = 0.065, *η*^2^ = 0.009, 95% (0.00, 0.03).^4^ The mean difference between the commission condition (*M* = 4.49, s.d. = 2.36) and control condition (*M* = 3.96, s.d.= 2.18) was 0.55 (Cohen’s *d* = 0.23). The equivalence test was significant, *t* (375.21) = −5.70, *p* < 0.001, given equivalence bounds of −1.82 and 1.82 and an alpha of 0.1. The 80% CI (0.24, 0.82) was completely within the equivalence bounds. For the rating distributions, see the electronic supplementary materials, plots for state memory distrust.

Similar results were found for the state distrust towards omission analysis. We did not detect a statistically significant main effect of the experimental condition, *F*_2, 617_ = 2.96, *p* = 0.053, *η*^2^ = 0.010, 95% (0.00, 0.03).^5^ The mean difference between the omission condition (*M* = 5.07, s.d. = 2.53) and control condition (*M* = 4.52, s.d. = 2.17) was 0.55 (Cohen’s *d* = 0.23). The equivalence test was significant, *t* (410.62) = −5.92, *p* < 0.001, given equivalence bounds of −1.89 and 1.89 (on a raw scale) and an alpha of 0.1. The 80% CI (0.26, 0.84) was completely within the equivalence bounds.

Looking at the data from another point of view, for all pairwise comparisons of the state distrust towards commission ratings between the commission condition and the control conditions, 50.83% of the time, the ratings from the commission condition were higher, while 37.13% of the time, the reverse was true. For all pairwise comparisons of the state distrust towards omission ratings between the omission condition and the control conditions, 50.58% of the time, the ratings from the commission condition were higher, while 38.09% of the time, the reverse was true, reaffirming that the effects of manipulation were smaller than we had hoped for. We therefore concluded that the manipulation did not reach an adequate strength in manipulating state memory distrust. Despite the manipulation not producing the intended effects, we proceeded with the main analyses to examine potential effects that may still inform our research question.

#### 7.1.2. The effect of feedback on response bias

To examine whether the experimental feedback manipulation would cause a shift in response bias, we first calculated the SDT response criterion indices c and *β* for the second recognition test (see table 2 for descriptive statistics). Recall that a higher value of c or *β* would indicate a more conservative response criterion (i.e. more likely to make ‘new’ judgements). Next, we performed one-way ANOVAs with the experimental condition (feedback: commission versus omission versus no feedback) as a between-participant factor and with c or *β* in the second test as the dependent variable, respectively. The effects of manipulations are reported averaging over manipulation check order conditions.^6^

**Table 2.** Descriptive statistics of the response criterion.

	*c*		*β*	
	mean	s.d.	mean	s.d.
control	0.16	0.46	1.70	1.44
feedback commission	0.40	0.48	2.32	1.84
feedback omission	−0.15	0.56	1.32	1.36

Results indicated that there was a large and statistically significant main effect of experimental conditions on criterion c, *F*_2,619_ = 60.06, *p* < 0.001, *η*^2^ = 0.16, 95% (0.11, 0.21). Criterion c was the highest (i.e. strictest) in the feedback-commission condition, followed by the control condition and the feedback omission condition. All pairwise comparisons were statistically significant (*p*s < 0.001). The equivalence test for the commission-control comparison was non-significant, *t* (381.47) = 2.98, *p* = 0.998, given equivalence bounds of −0.094 and 0.094, equal to a Cohen’s *d* = 0.2, and an alpha of 0.1. The 80% CI for the mean difference between the commission condition and the control condition was (0.17, 0.29), which lies entirely above the equivalence bounds. The equivalence test for the omission-control comparison was non-significant as well, *t* (401.98) = −4.40, *p* > 0.999, given equivalence bounds of −0.102 and 0.102 (equal to a Cohen’s *d* = 0.2) and an alpha of 0.1. The 80% CI for the mean difference between the commission condition and the control condition was (−0.38, −0.26), which lies entirely below the equivalence bounds.

As for the analysis on *β,* results showed that there was a statistically significant main effect of experimental conditions, *F*_2,619_ = 20.70, *p* < 0.001, *η*^2^ = 0.06, 95% (0.03, 0.10). Similarly, *β* was the highest in the feedback-commission condition, followed by the control condition and the feedback omission condition. All pairwise comparisons were statistically significant (*p*s < 0.026). The equivalence test for the commission-control comparison was non-significant, *t* (337.98) = 1.73, *p* = 0.958, given equivalence bounds of (−0.33, 0.33) and an alpha of 0.1. The 80% CI for the mean difference between the commission condition and the control condition was (0.40, 0.83), which lies entirely above the equivalence bounds. The equivalence test for the omission-control comparison was non-significant as well, *t* (435.95) = −0.78, *p* = 0.781, given equivalence bounds of (−0.28, 0.28) and an alpha of 0.1. The 80% CI for the mean difference between the commission condition and the control condition was (−0.60, −0.16), which partly overlaps with the equivalence region. Given that *β* is a ratio statistic and more prone to bias, we draw conclusions primarily based on the results of criterion c. The results, therefore, suggest that both forms of our experimental manipulation led to a significant criterion shift. As hypothesized, the response criterion was the most conservative in the feedback-commission condition, followed by the control condition. The feedback-omission condition had the least conservative (most liberal) response criterion.

Furthermore, we ran regression analyses on response criterion in the second test, with state memory distrust towards commission and omission errors being entered in the model separately. We did not find a significant association of criterion c with either state memory distrust towards commission (*B* = −0.002, SE = 0.01, *p* = 0.821) or state memory distrust towards omission (*B* = −0.003, SE = 0.01, *p* = 0.739). Likewise, neither state memory distrust towards commission (*B* = −0.02, SE = 0.03, *p* = 0.556) nor state memory distrust towards omission (*B* = −0.04, SE = 0.03, *p* = 0.114) was a significant predictor of *β*.

Taken together, these results suggest that the experimental manipulation mainly influenced the criterion. However, this effect on the criterion was likely not through the hypothesized effect on state memory distrust, assuming that our manipulation check measures were indeed measuring state memory distrust as intended. In the following section, we performed a few exploratory analyses to gain more insight into the current results.

## 8. Exploratory analyses

### 8.1. Effect of feedback on recognition, recollection and belief

To examine whether the experimental manipulation influenced recognition judgements, recollection and belief ratings in the second test, we ran linear mixed models (LMMs) using the lme4 package [40], each with one of these variables as the dependent variable and with the experimental conditions as fixed effects. We also included random intercepts for stimuli and participants in the models. These exploratory analyses showed that the experimental manipulations had an effect on recognition, recollection and belief judgements (see table 3). Compared to participants in the control condition, participants in the feedback-commission condition made fewer recognition judgements (i.e. ‘old’) and reported lower recollection and belief ratings, whereas participants in the feedback-omission condition made more recognition judgements and reported higher recollection and belief ratings, thus suggesting a calibration of metamemory judgements after receiving feedback.

**Table 3.** The effect of experimental manipulation on recollection, belief and recognition.

	recollection			belief			recognition		
fixed effects									
	*B*	SE	*p*	*B*	SE	*p*	*B*	SE	*p*
intercept	4.01	0.09	<0.001	4.21	0.08	<0.001	−0.20	0.06	0.001
commission versus control	−0.28	0.12	0.017	−0.46	0.10	<0.001	−0.31	0.07	<0.001
omission versus control	0.48	0.11	<0.001	0.46	0.10	<0.001	0.45	0.07	<0.001
random effects									
		s.d.	ICC		s.d.	ICC		s.d.	ICC
participants		1.13	0.16		0.93	0.12		0.61	0.10
stimuli		0.44	0.02		0.44	0.03		0.38	0.04
pseudo-*R*^2^ (fixed/total)	0.01/0.19			0.02/0.16			0.02/0.16		

ICC, Intraclass correlation.

We performed another analysis on the recognition judgements, including belief rating as a covariate. Results showed that after controlling for belief ratings, the effects of feedback manipulation became non-significant (commission: *B* = 0.06, SE = 0.22, *p* = 0.796; omission: *B* = 0.23, SE = 0.21, *p* = 0.258).

### 8.2. Correlation between state and trait memory distrust

Given that state memory distrust was not correlated with response criterion c, we investigated the validity of the state memory distrust items by examining their partial association with the SSMQ and MDS. Recall that the SSMQ primarily concerns errors of omission, whereas the MDS primarily concerns errors of commission; recall also that we reverse-scored the SSMQ such that high scores on both measures would signify higher levels of memory distrust. Our results showed that MDS scores positively predicted state memory distrust towards commission (*ß* = 0.35, SE = 0.04, *p* < 0.001), whereas SSMQ did not (*ß* = −0.07, SE = 0.04, *p* = 0.124). For state memory distrust towards omission, though, both MDS and SSMQ positively predicted the ratings (MDS: *ß* = 0.29, SE = 0.04, *p* < 0.001; SSMQ: *ß* = 0.17, SE = 0.04, *p* < 0.001). These results, therefore, suggest that the two manipulation checks did capture elements of memory distrust to an extent, but that they may not have been sufficiently selective in capturing the distinctive aspects of distrust towards commission or omission.

### 8.3. Correlation of state memory distrust with recollection and belief

To further explore the validity of the state memory distrust measures, we examined whether they predicted belief and recollection ratings. We ran LMMs with either recollection or belief rating in the second test as the dependent variable and state memory distrust towards commission errors and omission errors as fixed effects. We also included random intercepts for stimuli and participants in the models. State memory distrust towards commission errors positively predicted recollection ratings (*B* = 0.06, SE = 0.03, *p* = 0.018), while state memory distrust towards omission negatively predicted recollection ratings (*B* = −0.05, *SE* = 0.02, *p* = 0.037). No significant associations were found for either belief ratings (distrust commission: *B* = 0.03, SE = 0.02, *p* = 0.226; distrust omission: *B* = −0.02, SE = 0.02, *p* = 0.428) or recognition judgements (distrust commission: *B* = 0.004, SE = 0.02, *p* = 0.796; distrust omission: *B* = −0.002, SE = 0.02, *p* = 0.873). Therefore, in our data, people who considered themselves more likely to make commission errors at the present moment reported slightly higher recollection ratings, while people who considered themselves more likely to make omission errors at the present moment reported slightly lower recollection ratings only. The non-significant correlation between the state memory distrust measures and belief and recognition judgements could suggest that our hypothesized calibration was at work. For example, although people with higher state memory distrust towards commission had, on average, more recollective experiences compared with their low distrust counterparts, they could have taken into account their tendency to make memory errors and adjusted belief ratings downward, resulting in the non-significant relationship between state memory distrust and belief ratings.

Further analyses on belief and recognition judgements with recollection ratings included as a covariate showed that once conditioned on recollection, state memory distrust towards commission was negatively associated and state memory distrust towards omission positively associated with belief ratings (distrust commission: *B* = −0.03, SE = 0.01, *p* = 0.016; distrust omission: *B* = −0.03, SE = 0.01, *p* = 0.008) and recognition judgements (distrust commission: *B* = −0.16, SE = 0.05, *p* = 0.004; distrust omission: *B* = −0.03, SE = 0.05, *p* = 0.008), consistent with the calibration hypothesis. Note that the correlations were small, and the previous analysis showed that our state memory distrust measure may not be up to the task; we, therefore, caution against overinterpreting this result. Below, we further test the calibration with the established trait measures.

### 8.4. Correlation of trait memory distrust with recollection and belief

Because of the unexpected results regarding state memory distrust, we run exploratory analyses with trait memory distrust measures to examine potential calibration in reporting. First, we examined the correlation between trait memory distrust and recollection ratings. Results showed that the MDS positively predicted recollection, while the SSMQ negatively predicted recollection (see table 4), consistent with Zhang *et al*. [25]. Then we ran a second model for belief rating, with recollection rating added as a covariate. This model reveals that when conditioned on recollection rating, the MDS negatively predicted, while the SSMQ positively predicted belief ratings. Put differently, when recollection judgements are the same, people who considered themselves as prone to making commission errors tended to give lower belief ratings, while people who considered themselves as prone to making omission errors tended to give higher belief ratings. Similar results were found regarding recognition judgements. This again seems to corroborate our calibration hypothesis.

**Table 4.** The effect of trait memory distrust on recollection, belief, and recognition.

	recollection			belief			recognition		
fixed effects									
	*B*	SE	*p*	*B*	SE	*p*	*B*	SE	*p*
intercept	4.03	0.06	<0.001	0.44	0.08	<0.001	−7.58	0.12	<0.001
MDS	0.27	0.05	<0.001	−0.06	0.02	0.009	−0.62	0.09	<0.001
SSMQ	−0.36	0.05	<0.001	0.11	0.02	<0.001	0.59	0.09	<0.001
recollection				0.93	0.00	<0.001	1.87	0.02	<0.001
random effects									
		s.d.	ICC		s.d.	ICC		s.d.	ICC
participants		1.03	0.13		0.53	0.29		1.88	0.51
stimuli		0.43	0.02		0.06	0.00		0.38	0.02
pseudo-*R*^2^ (fixed/total)	0.01/0.17			0.88/0.91			0.80/0.91		

Recall that we explicitly asked participants whether they calibrated their responses in the experiment (on a 7-point scale). Participants on average gave a rating of 4.48 (s.d. = 1.76), and 60.45% of all participants gave a rating of 5 and above, indicating that the majority of them did take into account their (perceived) tendency to make memory errors. To further test this possibility of calibration, we included this calibration rating in the models predicting belief ratings and recognition judgements and had it interact with each of the two memory distrust measures (see table 5). Results showed that the interaction term between calibration rating and MDS was statistically significant as a predictor of belief ratings. Put differently, when having the same recollection, people who considered themselves more prone to commission errors and who took into consideration this tendency reported lower belief ratings. No other interaction terms were statistically significant. Additional analysis was performed to examine whether this self-reported calibration moderated response criterion c in the second test. We did not find a significant interaction effect on criterion c between calibration effort and experimental manipulation (calibration * commission versus control: *B* = 0.07, SE = 0.05, *p* = 0.139; calibration * omission versus control: *B* = 0.01, SE = 0.05, *p* = 0.771).

**Table 5.** Self-reported calibration effort and belief as well as recognition.

	belief			recognition		
fixed effects						
	*B*	SE	*p*	*B*	SE	*p*
intercept	0.46	0.02	<0.001	−7.56	0.13	<0.001
calibration	−0.04	0.02	0.083	0.03	0.09	0.751
MDS	−0.06	0.02	0.028	−0.63	0.09	<0.001
SSMQ	0.11	0.02	<0.001	0.59	0.09	<0.001
recollection	0.93	0.00	<0.001	1.87	0.02	<0.001
MDS * calibration	−0.11	0.02	<0.001	−0.11	0.09	0.234
SSMQ *calibration	0.02	0.02	0.345	0.08	0.09	0.377
random effects						
		s.d.	ICC		s.d.	ICC
participants		0.52	0.29		1.88	0.51
stimuli		0.06	0.00		0.38	0.02
pseudo-*R*^2^ (fixed/total)	0.88/0.91			0.80/0.91		

## 9. Discussion

In this experiment, we aimed to experimentally manipulate state memory distrust towards commission and omission separately and examine their effect on criterion shift. Although we observed hypothesized effects of the manipulation on criterion shift, we did not find our hypothesized effect of the manipulation on state memory distrust itself. As a matter of fact, our analysis showed that the experimental manipulation had a negligible effect on the state memory distrust measures. This may indicate that the feedback manipulation was not strong enough to alter participants’ memory distrust levels, or that the state memory distrust measure did not adequately capture the change in state memory distrust levels. In the following section, we discuss the results in relation to the literature.

### 9.1. Biased feedback effects on criterion shift

Expanding on previous research, which showed that biased positive feedback can shift participants’ response criterion in either a more lax or strict way [29], we showed that biased negative feedback can have a similar effect. Consistent with our expectations, the observed differences in criterion c between the feedback-commission and feedback-omission conditions in the current study were *c*_diff_ = 0.40 in the first test and *c*_diff_ = 0.55 in the second test, in between the effect sizes observed in prior studies using explicit instruction [31] and studies using biased positive feedback [29]. Equivalence tests showed that the observed effects were significantly larger than our SESOI, which corresponds to a Cohen’s *d* of 0.2. In fact, the point estimate of pairwise comparison between feedback-commission condition and feedback-omission condition, *c*_diff =_ 0.55, translates to Cohen’s *d* = 1.0. To put this effect size in more intuitive terms, there is a 76.0% chance that a person randomly selected from the feedback-commission group would have a stricter response criterion than a person randomly selected from the feedback-omission group (probability of superiority).

The large observed effect of the feedback manipulation on criterion shift is in stark contrast with the fact that we did not find the expected effect of the feedback manipulation on state memory distrust, even though the differences in state memory distrust between feedback conditions and control conditions were in the expected directions. Equivalence tests, however, showed that the effects were too small to be considered relevant. Furthermore, we did not find evidence that state memory distrust was associated with response criterion, further casting doubt on the hypothesized mechanism.

There could be several explanations for this surprising finding. The first explanation is that the experimental manipulation affected response criterion not through state memory distrust but through another mechanism, such as a conscious shift of response strategy given the feedback and the incentive to achieve high accuracy. That is, participants could have strategically responded to feedback on commission (omission) errors by choosing ‘new’ (‘old’) whenever their memory of the stimulus was ambiguous and uncertain. This adjustment of strategy does not necessarily require participants to doubt their memory. A second possibility is that including both aspects of memory distrust in the summary feedback might have introduced ambiguity, which unintentionally influenced participants’ responses to the manipulation checks. Third, it is possible that the feedback on performance *did* influence levels of memory distrust, but not on participants’ *global* memory appraisals. Since our manipulation check questions were framed quite generally (e.g. ‘At this moment, if I am asked to retrieve something from memory, I think I likely would remember things that did not happen’), it could be that participants’ answers were based on expectations about their memory performance, not restricted to the test format. In this case, if we were to measure participants’ state memory distrust with a more task-specific prompt such as ‘if I continue with this memory test, I think I likely would recognize photos that in fact were not in yesterday’s session’, then we might capture more precise assessments of state memory distrust that are more strongly influenced by the feedback. Third, although we deliberated on the item construction for the manipulation check questions, and they appear to have good face validity, it is possible that the participants interpreted them in a different way. The fact that state memory distrust towards omission was correlated more strongly with the MDS than with the SSMQ might indicate cause to speculate that some participants did not interpret the item as intended. Finally, the use of a single-item measure may have also suffered from low reliability, allowing too much noise in responses.

Despite these possible issues with the measure, exploratory analyses revealed some meaningful patterns. When recollection was controlled for, participants with higher (versus lower) state memory distrust towards commission errors tended to give lower belief ratings and were less likely to recognize an item as old. On the other hand, a reversed pattern is found regarding state memory distrust towards omission. Given these possibilities, we conclude that the current evidence cannot offer a clear picture of whether or not state memory distrust indeed played a role in the response criterion shift.

### 9.2. Evidence for calibration based on trait memory distrust measures

Trait memory distrust could be associated with metacognitive judgements through different mechanisms. First, trait memory distrust could be partly reflecting objective memory functioning. In this case, we would expect that, for example, people with high distrust towards commission errors indeed are more likely to have more vivid recollective experiences and therefore higher recollection and belief ratings. Second, when motivated, people can also calibrate their belief judgements based on their tendency of committing memory errors, leading people with high distrust towards commission to downregulate their belief ratings and people with high distrust towards omission to upregulate their belief ratings. Our data suggest that people who considered themselves as more prone in general to making commission errors reported, on average, the highest recollection and belief ratings, while the reverse was true for those with higher distrust towards omission errors. Further analysis also showed that, when conditioned on recollection ratings, people with higher distrust towards commission errors reported on average lower belief ratings, whereas those with higher distrust towards omission errors reported on average higher belief ratings. Taken together, the results suggest that people indeed can calibrate their response criterion when being incentivized; however, the overall effect is much smaller than anticipated.

### 9.3. Limitations and future directions

As discussed earlier, a major limitation of the current study is the lack of clarity over the extent to which we successfully manipulated state memory distrust. Our first attempt at manipulating the two aspects of memory distrust revealed less than optimal results. While we observed some differences in state memory distrust between conditions, the manipulation did not produce the expected changes in state memory distrust that would align with our hypothesized mechanism of criterion shift. In the recent work of Dudek & Polczyk [32], the authors also found some unexpected results regarding their memory distrust manipulation. Although they found significant differences in state memory distrust between conditions, this effect did not lead to an increase in susceptibility to misinformation as hypothesized. This suggests that memory distrust, particularly in an experimental context, may be more difficult to manipulate than previously thought. The aim to manipulate the two aspects of distrust separately further increased its difficulty. The disconnect between memory distrust and susceptibility to external influences highlights the need for more nuanced and effective manipulations. Based on these findings, we recommend future research to build on our work as well as that of others to further refine the manipulation procedures (e.g. measuring state memory distrust both pre- and post-manipulation) and establish better state memory distrust measures, such as a multi-item scale with established reliability and validity. Research on the effects of memory distrust on various memory phenomena needs a stronger foundation.

## 10. Conclusion

This study aimed to manipulate state memory distrust towards commission and omission separately and examine their effect on criterion shift. Our findings indicate that feedback manipulation did influence response criteria: participants who were told they frequently made commission errors adopted a stricter response criterion, while those who received feedback about omission errors shifted towards a more liberal criterion. However, we did not find sufficient evidence that our manipulations had the expected effect on state memory distrust. Therefore, we could not offer evidence that the effect of feedback on criterion shift was through memory distrust. Overall, these results highlight the potential for feedback to influence metacognitive control of memory, but also point to the limitations in effectively manipulating aspects of state memory distrust separately. More research is needed to refine and improve state memory distrust manipulation and measurement.

## Data Availability

All study materials are available at the OSF [41]. Supplementary material is available online [42].

## Appendix A. Feedback script

### Appendix A.1. Commission error condition

Thank you for completing the first memory test. We have collated your responses and evaluated your performance. In the previous test, while you were pretty good at correctly identifying old photos, in the meantime, you mistakenly recognized several new photos as old, indicating that these new photos were present in the first part of the study, while in reality, they were not. Your accuracy at *correctly identifying new photos* is among the lowest *20%* among all participants so far. That is, on average, you made more such errors than other participants tables 6 and 7.

**Table 6.** Descriptives of the synthetic dataset.

condition	*d*′	*c*	*β*	*n*_hit_	*n*_false alarm_
	M (s.d.)	M (s.d.)	M (s.d.)	M (s.d.)	M (s.d.)
commission	1.93 (0.69)	0.15 (0.31)	1.71 (1.37)	15.73 (2.61)	2.83 (2.42)
control	1.87 (0.64)	0.08 (0.29)	1.30 (0.64)	15.94 (2.61)	3.15 (2.38)
omission	1.86 (0.69)	0.03 (0.30)	1.18 (0.62)	16.14 (2.64)	3.49 (2.58)

**Table 7.** Analyses on stimuli valence and arousal ratings.

model	effect	*F* statistics	*p*‐value	*η* ^2^
arousal	old versus new	*F* (1, 76) = 1.27	0.263	= 0.016
	block	*F* (1, 76) = 0.04	0.850	<0.001
	old versus new * block	*F* (1, 76) = 0.70	0.407	= 0.009
valence	old versus new	*F* (1, 76) = 0.01	0.910	<0.001
	block	*F* (1, 76) = 1.25	0.267	= 0.016
	old versus new * block	*F* (1, 76) = 0.17	0.679	= 0.002

It is possible that you sometimes have difficulty distinguishing events you remember from those you only imagined. You sometimes may also become uncertain whether a recalled event really happened, or whether you saw it on TV or in a movie. Your ability to remember the source of information and the context of events may not be complete.

It’s important to note that participants who rank in the *top 10%* in the *second memory test* will receive a $3 bonus, and it is important to complete the experiment seriously.

## Appendix A.2. Omission error condition

Thank you for completing the first memory test. We have collated your responses and evaluated your performance. In the previous test, while you were pretty good at correctly identifying new photos, in the meantime, you mistakenly recognized several old photos as new too, indicating that these old photos were not present in the first part of the study, while in reality, they were. Your accuracy at *correctly identifying old photos* is among the lowest *20%* among all participants so far. That is, on average, you made more such errors than other participants.

It is possible that you often experience the tip-of-the-tongue phenomenon when you know that you remember something but are unable to express it. Sometimes, you may also find it difficult to recall what you were doing after you have taken your mind off it for a few minutes, especially under stress and pressure. In the future, memorizing things and successfully recalling them after a while may become more difficult.

It’s important to note that participants who rank in the *top 10%* in the *second memory test* will receive a $3 bonus, and it is important to complete the experiment seriously.

## Appendix A.3. Control condition

Thank you for completing the first memory test. We have gathered your responses.

This is a 1 min break before the second memory test commences.

Please keep in mind that people who rank *top 10%* in the *second memory test* will get a $3 bonus, and it is important to complete the experiment seriously.

^1^
In reality, the probabilities will likely not be symmetrical. However, we consider the potential effect of the differences of probabilities on estimates insubstantial, given that we set a rather conservative SESOI.
^2^
In Zhang *et al*. (2023)[27], the trait memory distrust measure and memory task were measured three days apart, and their correlation was *r* = 0.19. In the current study, the association is expected to be stronger given that we will measure state memory distrust right before or after the memory task and plan to increase participants’ motivation to be accurate by raising the performance bonus to $3, 100% of the basic experiment payoff.
^3^
In each data collection cycle, the time window for participating in session 1 was 3 h. For the second session, we expected a long time (e.g. 6 h) to complete data collection, given the pool would be restricted to participants who had signed up for session 1. The potential range of gap between sessions 1 and 2 therefore was 21 (24 − 3) hours to 30 (24 + 6) hours.
^4^
We ran an exploratory analysis with manipulation check order as a factor, which did not find evidence that the timing of the manipulation check had a main effect, *F*_1,616_ = 0.84, *p* = 0.361, *η*^2^ = 0.001, 95% (0.00, 0.01) or that it interacted with experimental conditions, *F*_2,616_ = 1.03, *p* = 0.359, *η*^2^ = 0.003, 95% (0.00, 0.02).
^5^
Exploratory analysis with manipulation check order as a factor did not show evidence that the timing of the manipulation check had a main effect, *F*_1,614_ = 0.48, *p* = 0.488, *η*^2^ = 0.001, 95% (0.00, 0.01) or that it interacted with experimental conditions, *F*_2,614_ = 1.32, *p* = 0.267, *η*^2^ = 0.004, 95% (0.00, 0.02).
^6^
Exploratory analysis showed that the effects of feedback manipulation on response bias were larger among conditions where participants completed the manipulation check after (versus before) the second recognition test. However, the moderation effect did not reach statistical significance, *F*_2,616_ = 2.84, *p* = 0.059, *η*^2^ = 0.009, 95% CI (0.00, 0.03).
